# Supplementary material for: Identification and functional analysis of non-coding regulatory small RNA FenSr3 in Bacillus amyloliquefaciens LPB-18
Source: PeerJ. 2023 May 15;11:e15236. doi: 10.7717/peerj.15236 (PMC10194069; doi:10.7717/peerj.15236)
Supplement: Supplemental Information 4 [file peerj-11-15236-s004.zip › KO/CK-vs-T1_map/map04971.html]

KEGG PATHWAY: Gastric acid secretion - Reference pathway


|  |  |
| --- | --- |
| **Gastric acid secretion - Reference pathway** |  |

[
Pathway menu
| Organism menu
| Pathway entry
| Show description
| User data mapping
]

Gastric acid is a key factor in normal upper gastrointestinal functions, including protein digestion and calcium and iron absorption, as well as providing some protection against bacterial infections. The principal stimulants of acid secretion at the level of the parietal cell are histamine (paracrine), gastrin (hormonal), and acetycholine (ACh; neurocrine). Stimulation of acid secretion typically involves an initial elevation of intracellular calcium and cAMP, followed by activation of protein kinase cascades, which trigger the translocation of the proton pump, H+,K+-ATPase, from cytoplasmic tubulovesicles to the apical plasma membrane and thereby H+ secretion into the stomach lumen.

|  |  |  |
| --- | --- | --- |
| Reference pathway | 184% 150% 122% 100% 82% 67% 55% | 图片下载 |
